# Genetic characterization of *Plectorhinchus mediterraneus* yields important clues about genome organization and evolution of multigene families

**DOI:** 10.1186/1471-2156-13-33

**Published:** 2012-04-30

**Authors:** Manuel A Merlo, Silvia Portela-Bens, Ismael Cross, Manuel Manchado, Laureana Rebordinos

**Affiliations:** 1 Laboratorio de Genética, Universidad de Cádiz, Polígono Río San Pedro, 11510, Puerto Real, Cádiz, Spain; 2 ICMAN – CSIC, Polígono Río San Pedro, 11510, Puerto Real, Cádiz, Spain; 3 Centro IFAPA, Junta de Andalucía, El Toruño, Cádiz, 11500, Spain

## Abstract

**Background:**

Molecular and cytogenetic markers are of great use for to fish characterization, identification, phylogenetics and evolution. Multigene families have proven to be good markers for a better understanding of the variability, organization and evolution of fish species. Three different tandemly-repeated gene families (45S rDNA, 5S rDNA and U2 snDNA) have been studied in *Plectorhinchus mediterraneus* (Teleostei: Haemulidae), at both molecular and cytogenetic level, to elucidate the taxonomy and evolution of these multigene families, as well as for comparative purposes with other species of the family.

**Results:**

Four different types of 5S rDNA were obtained; two of them showed a high homology with that of *Raja asterias*, and the putative implication of a horizontal transfer event and its consequences for the organization and evolution of the 5S rDNA have been discussed. The other two types do not resemble any other species, but in one of them a putative tRNA-derived SINE was observed for the first time, which could have implications in the evolution of the 5S rDNA. The ITS-1 sequence was more related to a species of another different genus than to that of the same genus, therefore a revision of the Hamulidae family systematic has been proposed. In the analysis of the U2 snDNA, we were able to corroborate that U2 snDNA and U5 snDNA were linked in the same tandem array, and this has interest for tracing evolutionary lines. The karyotype of the species was composed of 2n = 48 acrocentric chromosomes, and each of the three multigene families were located in different chromosome pairs, thus providing three different chromosomal markers.

**Conclusions:**

Novel data can be extracted from the results: a putative event of horizontal transfer, a possible tRNA-derived SINE linked to one of the four 5S rDNA types characterized, and a linkage between U2 and U5 snDNA. In addition, a revision of the taxonomy of the Haemulidae family has been suggested, and three cytogenetic markers have been obtained. Some of these results have not been described before in any other fish species. New clues about the genome organization and evolution of the multigene families are offered in this study.

## Background

Multigene families are groups of genes which are functionally related and show similar sequences, having descended from a common ancestral gene [1]. Concerted evolution is the model which has commonly been used to explain the long-term evolution of these multigene families. Under this evolution model the gene array undergoes homogenizing forces, such as unequal crossing-over, gene conversion or purifying selection [2], that tend to remove or to spread the mutations which arise in a gene unit from the family. However, although the evolution of some multigene families fits this model, there are other families which do not present a good fit with concerted evolution. The histone family (H1, H2A, H2B, H3 and H4) is an example [1], since their coding regions are similar, owing to the purifying selection mechanism, but spacer sequences are highly variable. The evolution model which explains this situation better is the birth-and-death model, under which new genes are originated by successive duplications, and these new genes are either maintained for a long time or lost, or degenerate into pseudogenes. It has traditionally been thought that nuclear ribosomal DNA (45S and 5S) and U2 small nuclear RNA (snRNA) genes are multigene families that undergo a concerted long-term evolution. In recent years, however, it has been discovered that some species present a birth-and-death evolution model in these multigene families; these include filamentous fungi [3], razor clams [2,4] and some fish species [5,6]. Despite this, in aquatic organisms, rDNA multigene families are of great interest for species characterization and discrimination between closely-related species [7]. Several phylogenetic analyses have also focused on sequences of the rDNA genes [8]. At the cytogenetic level the ribosomal RNA genes have been shown to be useful tools for providing chromosome molecular markers [9], for analyzing the evolution processes of species [10] and for phylogenetic analysis and species identification [11].

The small nuclear RNA (Us snRNA) gene families have scarcely been studied at molecular and cytogenetic levels. The U1 snRNA has been characterized in human [12], mouse [13], fish [14], and crustaceans [15,16]. Similarly, the U2 snRNA gene family has been studied in humans [17,18], primates [19], and fishes [9,11,20]. The U2 snRNA gene appears to be governed by the concerted evolution model [1,21], although more studies are needed to confirm this affirmation definitively.

*Plectorhinchus mediterraneus* is a demersal fish from the Haemulidae family whose habitat extends along Eastern Atlantic coasts, from Portugal to Namibia, including the Canary Islands and Western Mediterranean coasts [22]. This species is of commercial interest in Mediterranean countries, where there is significant consumer demand alongside other better-known species such as *Sparus aurata* (Sparidae), *Pagrus pagrus* (Sparidae), *Pagrus auriga* (Sparidae), and *Argyrosomus regius* (Sciaenidae). Experimental work has been carried out in *P. mediterraneus* as a species suitable for aquaculture [23].

The main objectives of this work are to provide new insights about the organization and evolution of multigene families, and to increase the scarce molecular and cytogenetic data on the Haemulidae family. To this end, a molecular characterization study has been made of the 5S rRNA gene the ITS-1 region of the major ribosomal gene, and the U2 snRNA gene of the species *Plectorhinchus mediterraneus*. Further, the 18S rRNA gene, 5S rDNA and U2 snRNA gene have been used as cytogenetic probes in Fluorescent *in situ* Hybridization (FISH) experiments, and these results have been compared with those obtained in other Haemulidae species.

## Results

### Analysis of the sequences

Up to six specimens were subjected to amplification with each primer pair. The PCR amplification with primers for the 5S rDNA (including the NTS) yielded a main electrophoretic band of 750 bp in all six specimens mentioned. Additional faint bands were observed whose sizes were 400, 290 and 200 bp. The PCR amplification with the ITS-1 and the U2 snRNA gene only yielded one band of 850 and 1100 bp, respectively.

Thirteen clones were sequenced from the 750 bp (type α hereafter) product of the 5S rDNA (including the NTS). Up to twelve additional clones were sequenced, five from the 400 bp product, one from the 290 bp and six from the 200 bp. After sequencing, it was confirmed that the 400 bp product was really a dimeric form of the 200 bp product. The 290 bp clone was named as type β. Two out of the six clones from the 200 bp product were different in sequence with respect to the other four clones, so they were divided into two types, named type γ and type δ respectively. All monomers obtained from the five 400 bp clones belonged to type γ. One of these monomers presented a 10 bp deletion in its coding region (from base +38 to +47).

**Figure 1:**
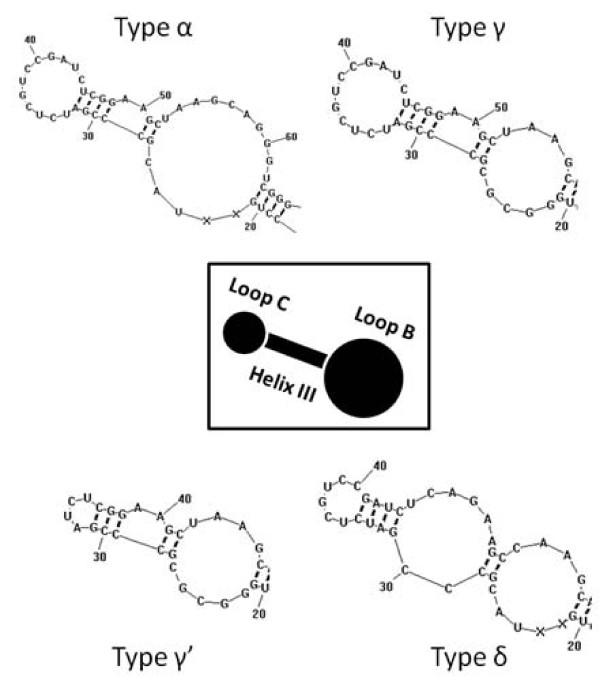
**Secondary structure of the 5S rRNA domain β.** 5S rRNA secondary structure from the different types obtained. Only Loop B, Helix III and Loop C are represented. A schematic representation of this region is in the center of the image. The β type did not produce any secondary structure in RNAstructure software. The γ’ type corresponds to the deleted form of this type.

Looking into the sequences, it could be ascertained that coding regions from the different types showed some variability among them (Table 1). Surprisingly, the evolutionary distances in this region were high in type β and moderate in type δ. Because of this, the secondary structure of type β and δ were examined, together with the remaining types (Figure 1), in order to determine whether they might be pseudogenes. The *RNAstructure v5.2* program was unable to obtain any secondary structure in the type β clone, and in one of the two clones of the δ type. The remaining clone of the δ type presented an abnormal secondary structure in the Helix III-Loop C region, compared with models described in the literature [24]. The secondary structure was also predicted for the monomer of the γ type with a 10 bp deletion, and this affects the Loop C size, making it smaller.

**Table 1:** 5S rRNA polymorphism

	α	β	γ	δ
**α**	**0 ± 0**	0.142	0.021	0.078
**β**	0.038	**-------**	0.157	0.154
**γ**	0.011	0.039	**0.016 ± 0.002**	0.091
**δ**	0.026	0.038	0.027	**0.053 ± 0.025**

Evolutionary distance (K, upper diagonal) and standard deviation (lower diagonal) among the different types of 5S coding regions. The nucleotide variability (π, bold values along the diagonal) within each type is also shown.

**Figure 2:**
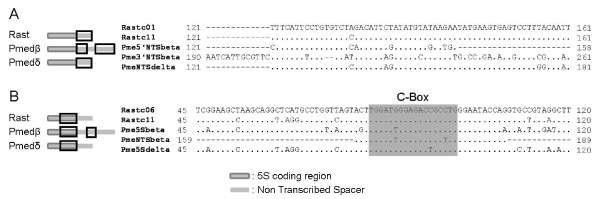
**5S rDNA homology between*Plectorhinchus mediterraneus*and*Raja asterias.*** Schematic representation (left) and alignment (right) in NTS (**A**) and coding regions (**B**) of *Raja asterias* and *Plectorhinchus mediterraneus*. The boxes in the schematic representation show the aligned region. Rast: *Raja asterias*; Pmed: *Plectorhinchus mediterraneus.*

The NTS of the β and δ types were subjected to a BLASTN search at the NCBI website (Figure 2A): 61 bp of the δ type NTS share homology with the NTS from two clones of *Raja asterias* (Acc. No. DQ020521.1 and DQ020520.1; E-value = 1·10^-15^ and 5·10^-14^, respectively). The same fragment was localized at the 3’ end of the NTS type β, and a smaller fragment of this (38 bp) was also observed at the 5’ end. Between the 5’ and 3’ ends a 31 bp fragment was found, corresponding to the C box of a 5S coding region. The 5S rDNA coding regions from β and δ types were also subjected to BLASTN search, excluding the primer binding sites. As expected, this region matches well with 5S rRNA sequences from many fish species but, curiously, the best fit was with those of *R. asterias* (Acc. No. DQ020548.1 and DQ020551.1; E-value = 1·10^-24^ and 2·10^-14^, respectively). The alignment of these coding regions and the C box fragment found in the type β NTS demonstrate the homology among these sequences (Figure 2B).

**Figure 3:**
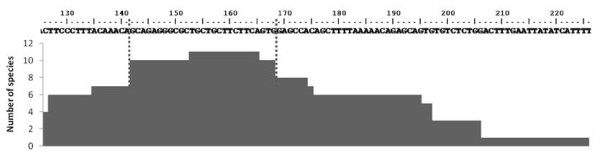
**Putative regulatory element inside NTS type α.** The 5’ end of the *Plectorhinchus mediterraneus* NTS type α. The graphic shows the number of species which share the same region in their NTS. The region most shared is highlighted between dotted lines.

**Figure 4:**
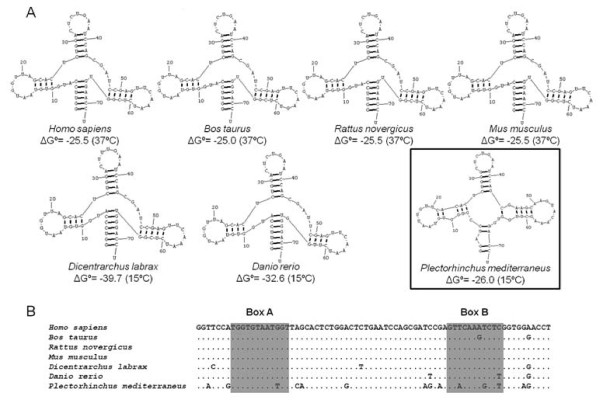
**tRNA-Gln secondary structure and sequence alignment.** Secondary structure (**A**) and alignment (**B**) of the tRNA-Gln from a variety organisms of belonging to mammals and fishes. The structures were predicted by RNAstructure 5.2 software, and different temperatures were entered depending on whether the organism is mammal (37°C) or fish (15°C).

The remaining NTS types were also submitted to BLASTN search. Three regions could be distinguished in type α. The first comprises 101 nucleotides (nt) of the 5’ end, and which is homologous with NTS regions from several different fish species (Figure 3). Further upstream from this site, both BLASTN and CENSOR programs detected a region similar to the glutamine tRNA gene; this sequence was isolated to be folded in both tRNAscan-SE and RNAstructure 5.2 programs. The first program could not fold any structure, but the second was able to do so, and this structure was compared with those from a variety of organisms (Figure 4A), and a different secondary structure was obtained with respect to those from different species of mammals and fishes; the stability of the molecule was less than that from the other fish species. Up to 14 polymorphic sites were observed in the *P. mediterraneus* sequence by aligning the tRNA sequences of all these organisms; four of these polymorphisms were in the most conserved regions (Box A and Box B), whereas the remaining species accumulated, at most, three polymorphic sites (Figure 4B). Thirdly, downstream from the tRNA sequence we were able to distinguish an AT-rich region (%AT = 77.18) of 149 bp in length, followed by a poly-A-like region (AAAAGGACAAAA). The NTS type γ did not show any homologies in the BLASTN search.

The PCR amplification with the ITS-1 primers yielded one electrophoretic band of 884–886 bp, of which, after sequencing, the first 185 bp were the 18S rRNA gene, followed by 608–610 bp belonging to the ITS-1 region and, finally, 91 bp of the 5.8S rRNA gene. The GC content of ITS-1 was very stable among the different clones (68.04%-68.47%). When the ITS-1 sequence was submitted to BLASTN, two main outputs could be observed, and both also belonged to the Haemulidae family (*Parapristipoma trilineatum* and *Plectorhinchus cinctus*), but the ITS-1 of *P. mediterraneus* was more similar to that of *Parapristipoma trilineatum* (E-value = 9·10^-178^) than to that of the same genus (E-value = 3·10^-146^).

**Figure 5:**
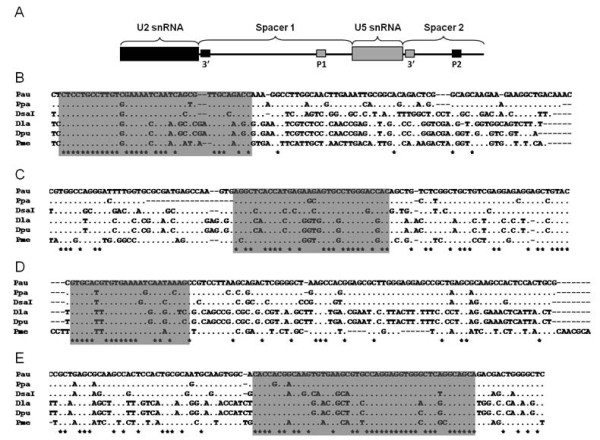
**U2 snRNA gene structure.** Schematic representation of the U2-U5 snDNA cluster (**A**). The 3’ box and PSE of the U2 snRNA gene (**B** and **E** respectively) and of the U5 snRNA gene (**C** and **D** respectively), are detected by alignment with other fish sequences (gray boxes). Key: 3’: 3’ box; P1: PSE of U5 snRNA gene; P2: PSE of U2 snRNA gene; Pau: *Pagrus auriga*; Ppa: *Pagrus pagrus*; Dsa: *Diplodus sargus*; Dla: *Dicentrarchus labrax*; Dpu: *Dicentrarchus punctatus*; Pme: *Plectorhinchus mediterraneus.*

The U2 snDNA yielded one PCR product of 1005–1052 bp. The reason for such length variability is the presence of several indel (insertion-deletion) regions. The spacer region was also submitted to a BLASTN search, and the U5 snRNA gene was found inside the region, showing a linkage between U2 and U5 snRNA genes. The 5’ and 3’ ends of the two spacers, which are generated by the presence of the two genes, were aligned with those from fish species available in the GeneBank Database (Figure 5). Thus, the 3’ box of the U2 snRNA gene was detected by aligning the 5’ end of the spacer between U2 and U5 genes (spacer 1) (Figure 5B); the PSE of the U5 snRNA gene by aligning the 3’ end of the same spacer (Figure 5C); the 3’ box of the U5 snRNA gene by aligning the 5’ end of the spacer between the U5 and U2 genes (spacer 2) (Figure 5D); and, finally, the PSE of the U2 snRNA gene by aligning the 3’ end of this last spacer (Figure 5E).

### Variability analysis

The types α, γ and δ of the 5S rDNA were used for comparative purposes with the other multigene family sequences. The DNA polymorphism analysis demonstrated that the 5S rDNA type γ and the region comprising the 18S rRNA (partial)-ITS-1-5.8S rRNA (partial) were the least variable, while the 5S rDNA type δ and the U2-U5 snDNA were the most variable (Table 2). The 5S type δ coding region showed a surprising high variability comparing with other coding regions. The analysis region-by-region showed that NTS type γ and NTS type δ are less variable than their respective coding regions. The spacer 1 of the U2-U5 was the region with more polymorphic sites and, together with the NTS type α region, presented the largest number of haplotypes. Moreover, the 5S rRNA type α, 5.8S rRNA genes and NTS type γ have no polymorphic sites among the clones of these genes.

**Table 2:** Gene polymorphism by gene region

**Region**	**Length (bp)**	**N**	**S**	**h**	**π**
*5S rRNA* (α)	120	13	0	1	0.000 ± 0.000
*NTS* (α)	627-650	13	19	11	0.009 ± 0.001
*5S rRNA* (γ)	110-120	14	1	2	0.002 ± 0.001
*NTS* (γ)	72	14	0	1	0.000 ± 0.000
*5S rRNA* (δ)	120	2	6	2	0.053 ± 0.025
*NTS* (δ)	61	2	1	2	0.017 ± 0.008
*18S rRNA*	185	14	3	4	0.002 ± 0.001
*ITS-1*	608-610	14	10	10	0.003 ± 0.001
*5.8S rRNA*	91	14	0	1	0.000 ± 0.000
*U2 snRNA*	189	13	6	6	0.007 ± 0.001
*Spacer 1*	533-570	13	52	11	0.031 ± 0.004
*U5 snRNA*	115	13	6	7	0.009 ± 0.002
*Spacer 2*	168-178	13	11	9	0.010 ± 0.002

N: number of sequences; S: number of polymorphic sites; h: number of haplotypes; π: nucleotide variability.

### Cytogenetic analysis

**Figure 6:**
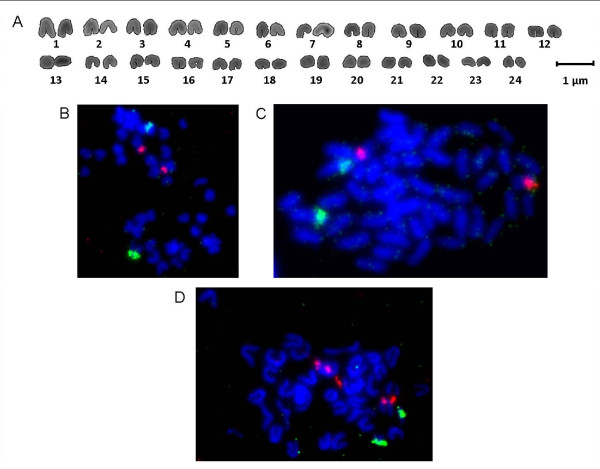
**Karyotype and Fluorescence*in situ*Hybridization in*Plectorhinchus mediterraneus.*** Karyotype of *P. mediterraneus* composed of 24 acrocentric chromosome pairs (**A**). Double-FISH treatment to localize simultaneously the 5S rDNA (red) and 18S rRNA genes (green) (**B**); U2-U5 snRNA gene (red) and 18S rRNA gene (green) (**C**); and U2-U5 snRNA gene (red) and 5S rDNA (green) (**D**).

The karyotype of *P. mediterraneus* presented a modal number of 48 acrocentric chromosomes (FN = 48) (Figure 6A), whose sizes were very small. Therefore, adjacent pairs of chromosomes were almost indistinguishable from each other by size.

Figures 6B, 6C and 6D respectively show the two colour-FISH results with the 18S rRNA gene and 5S rDNA probes, the 18S rRNA and U2-U5 snRNA gene probes, and the 5S rDNA and U2-U5 snRNA gene probes. None of the probes was co-localized with any other; thus three chromosomal markers were obtained. The 5S probe was localized in the subcentromeric position of one small chromosome pair. The 18S probe hybridized in the subcentromeric position of one of the largest chromosome pairs. Meanwhile, the U2-U5 probe also shows one signal in the subcentromeric position, in a medium-size chromosome pair.

## Discussion

The presence of 5S variants in the same genome has been described in several mammals, amphibians and fishes [6,25,26] among others, and this suggests that variant 5S types originated early in the evolution of the vertebrates [27]. Two types of 5S rDNA were also described in the *Leporinus* genus, and each was localized in a different locus, suggesting a separated evolution of the two types [28]. However, the presence of two 5S rDNA variants is common in fishes [29-31], and this feature has been attributed to the ancient duplication event that occurred before the divergence of the main groups of teleost fishes [32]. The presence of two variants in fishes has often been associated with a dual system where one of these variant is expressed in both somatic and oocyte cells, and the other variant only in oocyte cells, providing an increased physiological plasticity [33].

In the present work, up to four 5S rDNA variants were obtained (named from type α to type δ), which were characterized by their different NTS. The high evolutionary distance observed in the coding region of β and δ types leads to the hypothesis that they could be pseudogenes. The secondary structures of all 5S rRNA types (except β type and one clone of the δ type) showed secondary structures according to the models described [24]. The abnormal structure observed in the other clone of the type δ at the Helix III-Loop C region might be deleterious, since mutations occurring in this region could have lethal effects or affect the translation accuracy [34]; therefore it is possible that the β and δ types of 5S coding region could be pseudogenes. Similarly, the clone of the γ type with a deletion which affects the Loop C size could also be a non-functional gene.

The birth-and-death model has been used to explain the evolutionary mechanism of the 5S rDNA in several organisms such a fungal species [3], razor clam species [2], and fish species [6]. In the crustacean genus *Pollicipes*, up to seven different types of 5S rDNA and two pseudogenes have been described, suggesting a birth-and-death evolution pattern [35]. Initially, the birth-and-death model seems to drive the evolution of the 5S rDNA in *P. mediterraneus*, because variants and pseudogenes have been seen. However, is this the only model which acts in the 5S evolution? As mentioned above, the β and δ types showed a striking homology with 5S rDNA from *R. asterias*, in both the coding and NTS regions. *P. mediterraneus* and *R. asterias* are fishes which belong to different classes (Actinopterygii and Chondrichthyes respectively), which separated 527 My ago [36]. Two hypotheses can be outlined in this case: first, the β and δ types could have originated before the separation of the two classes, and could have been maintained in some lineages and lost in others. Nevertheless, the NTS are considered very dynamic regions of the genome, since they are free to mutate, and the variants which arise are almost neutral to natural selection; and they can be either fixed or lost, thus causing differences between closely related species, and even within individuals [37]. The pseudogenization of the β and δ types would accelerate the accumulation of mutations in the NTS region, making this first hypothesis unlikely. A second hypothesis should not be discounted: a Horizontal Transference (HT) event. After HT occurs, the transferred gene either maintains its functionality and stays in the host genome, or loses its functionality and becomes a pseudogene. The β and δ types of the *P. mediterraneus* 5S rDNA seem to be in this second situation. Therefore, before the HT happened, *P. mediterraneus* had two types of 5S rDNA (types α and γ), as described for many fish orders [31]. A process of duplication and specialization could have originated the types α and γ, as the birth-and-death model predicts, but within each type there is considerable homogeneity, as alignment within each type demonstrates. Hence, a mixed process could have driven the evolution of the 5S rDNA in *P. mediterraneus*, with the birth-an-death model acting at genome level and concerted evolution acting at locus level. This dual process has already been proposed for other fish species [38], and for the mollusc *Mytilus* spp. [8].

The precise mechanism by which the HT occurs is not yet well-understood, but there are two plausible mechanisms. One of these is mediated by transposable elements (TE) (see [39] for a review). The 5S rDNA family is an excellent candidate for this kind of HT, since interactions between retro-transposons and 5S rDNA have been demonstrated [40,41], and this interaction could be “the door” for a 5S rRNA gene lateral transfer. Another possible mechanism should be considered: sperm-mediated gene transference (SMGT), since it is well-known that sperm cells are able to capture exogenous DNA and to transfer it to the oocyte at fertilization [42]. The exogenous DNA is captured from the water column and the sediments, in which exogenous DNA is known to accumulate [43]. This, taken together with the external fecundation of the fish species, makes this HT mechanism a possibility. An HT event could have great evolutionary consequences as a source of biological innovation; indeed it has been postulated that the origin of the primordial eukaryote cell or some characteristics of multicellularity have developed from HT events [39].

The type α presented its own peculiarities, like the NTS fragment widely represented in the NTS of other fish species. In this case, up to 11 species from several fish families share 27 bp of this region (see Figure 3). This sequence could have some regulatory function as the enhancer element like that of the mammals, named the D-box, which can increase the transcription up to 10 times [44]. Also found within the same NTS fragment was an A-rich region (5’-CAAACAG-3’) similar to that found by [31] in some fish species (5’-GAAACAA-3’); those authors proposed this region as a second terminator region. The α type NTS also contains downstream another region very similar to the tRNA-Gln gene of various species. However, the *P. mediterraneus* secondary structure obtained with the RNAstructure program was different with respect to previously described models [45]; this, together with the higher accumulation of polymorphic sites in the *P. mediterraneus* tRNA sequence, leads to the proposal of the tRNA-Gln gene as a non-functional gene.

The 5S rDNA is known to be a multigene family which can be found linked with a variety of other multigene families, such as the major ribosomal genes, the histone genes, the trans-spliced leader genes, and the small nuclear RNA genes [9,46,47]; this indicates that the 5S rDNA has the ability “to jump” to other loci. This is not the first evidence of a 5S rDNA-tRNA linkage, since this kind of association has been found in a fungus species [48] and in various mussel species [8]. Here we describe for first time this kind of linkage in a fish species.

The tRNA presented here could be a short interspersed element (SINE). The majority of SINEs derive mainly from tRNA sequences [49] and share some common features: i) a tRNA-like region with slightly modified A box and B box regions; ii) a tRNA-unrelated region; iii) an AT-rich region ending with poly-A [50,51]. All these features are present in the type α NTS of *P. mediterraneus*, so it is possible that a tRNA-derived SINE is linked with the 5S rDNA type α. From an evolutionary point of view, it has been postulated that SINE integration into new localizations has potential implications, since it can disturb gene expressions or serve as a source of genomic innovation and a factor of genome plasticity [52].

The chromosomal localization of the 5S rDNA probe observed in *P. mediterraneus* (internal and near the centromere) is the most common pattern observed within fish species, so this could be the optimal localization for the arrangement of this gene [53]. The 5S rDNA localization has been determined in six species of the Haemulidae family, and between two and four chromosomes showed positive signals [54-56]. Therefore, in the Haemulidae family, some species could have suffered translocation events which have led to the gain of an additional 5S rDNA locus. The translocation to new chromosomal loci has traditionally been explained by unequal crossing-over between non-homologous chromosomes or by a transposon-mediated mechanism [57]. The signals from these six Haemulidae species were located either in telomeric position (in all species) of the q arm or near the centromere (in 4 out of the 6 species). The first location could be the pleisiomorphic condition in the Haemulidae family, which has been lost in *P. mediterraneus* and has retained the more conservative pattern of the 5S rDNA localization.

The ITS-1 size and GC content were very close to the average values for the Osteichthyes group (635.1 bp and 68.0% respectively) [58]. Two species of the Haemulidae fish family showed ITS-1 sequences particularly similar to that of *P. mediterraneus*. The ITS-1 region has secondary structures necessary for the rRNA maturation process, which give this region an intermediate rate of evolution [59]. These features make the ITS-1 region suitable for phylogenetic studies among closely-related groups [60]. Considering that *P. mediterraneus* showed an ITS-1 region more similar to that of *Parapristipoma trilineatum* than to that of the same genus, *Plectorhinchus cinctus*, a phylogenetic revision of the Haemulidae family should be addressed.

At the cytogenetic level, the 18S rRNA gene probe did not conserve the proposed plesiomorphic localization for fish species, i.e. one chromosome pair with signals in the telomeric position nearest to the centromere. This affirmation has been made based on Ag-NOR studies [61], but other studies using FISH techniques with major ribosomal probes have also confirmed it [10,11,57,62-65]. To the contrary, *P. mediterraneus* presented a derived (apomorphic) pattern in which the major ribosomal probe hybridizes in the subcentromeric region. Moreover, this localization is common in all Haemulidae species studied so far using both Ag-NOR staining and rDNA-FISH methods [54–55, and references therein]. Therefore, the pattern shown by *P. mediterraneus* is the plesiomorphic condition within the Haemulidae family. The non-syntenic arrangement of both ribosomal clusters, as we have found in *P. mediterraneus*, is the most commonly-observed situation in fishes [31]. The transcription by different RNA polymerases has traditionally been given as the explanation for this lack of co-localization [31]. Moreover, the separated localization could prevent undesirable disruptive translocations of the 5S rDNA into the major ribosomal cluster [64].

The U2 snRNA gene has been found linked to another snRNA: the U5 snRNA gene. This seems to be the common situation among the fishes so far studied with this gene family, since the same linkage has been seen in two species from the Moronidae family [11], four from the Engraulidae family [66] and in *Oreochromis niloticus*[14], and the U1, U2 and U5 snRNA linkage has also been observed within the Soleidae family [9,67]. In *Drosophila* species, different types of linkages have been observed, such as U1-U2, U2-U5 and U4-U5 linkages [68]. The linkage of different members of the snRNA gene family could serve as a means of consolidating phylogenetic lineages; indeed [67] were able to prove that U1-U2 linkage was present in the Soleidae family but not in the Scophthalmidae and Pleuronectidae families.

Moreover, by aligning both 5’ and 3’ ends of the two U2-U5 spacers with those from other fish species, it was possible to find four conserved regions which may be related with the 3’ box and the proximal sequence element (PSE) of the two genes. The 3’ box is necessary for the transcription termination and for the correct processing of the nascent mRNA [68], and is positioned at between 9 and 19 nucleotides (nt) downstream from the coding region [16]. The two 3’ boxes found in *P. mediterraneus* are a little nearer to the coding region. The PSE is essential for the initiation of transcription and is found between 40 and 60 nt upstream from the transcription starting point [69,70]; this is where the two PSE found in *P. mediterraneus* are situated. A third element, the distal sequence element (DSE), is normally located between 200 and 250 nt upstream from the PSE [71]. In the case of *P. mediterraneus*, several putative octamer motifs similar to that proposed for all vertebrate DSE [72] have been found (data not shown), but none of them are located at the cited nt range upstream from the PSE. The presence of all these regulator elements makes the U2 and U5 snDNA transcriptionally active genes.

The chromosome localization of the U2 snRNA gene has scarcely been studied in the fish group. In two species from the Moronidae family this probe hybridizes in the telomeric region of one acrocentric pair [11], and in four species from the Batrachoididae family it hybridizes in three different manners: dispersed, located in the subtelomeric position of one acrocentric pair, and both dispersed and located [10]. None of these cases share the same hybridization pattern as that observed in *P. mediterraneus*, and this makes the U2 snRNA gene of particular interest for cytotaxonomic purposes. To date only one case has been reported in fishes of co-localization between snRNA and ribosomal genes by means of the FISH technique, as is found in *Thalassophryne maculosa* (Batrachoididae family) [10]. Moreover, in *Solea senegalensis*, the 5S rDNA and the U1, U2 and U5 snDNA were found closely linked [9]. However, the non-syntenic organization of snRNA and ribosomal genes also seems to be the most common arrangement in fishes studied so far.

The spacer 2 of the U2-U5 cluster showed an exceptional low value of nucleotide variability with respect to the spacer 1 and the U2-U5 coding regions, and this peculiarity may be due to the small size of the spacer 2 region (168–178 bp). This size could be close to the minimum necessary for maintaining the U2 array and so only small variations can be afforded, in a similar way as had previously been proposed for short 5S arrays [31]. Similarly, the NTS type γ also presents low variability because the external promoters are included in a short NTS of 72 bp, and only slight variations should be tolerated. However, the same maintenance does not appear to occur in the NTS type δ, since its size is 61 bp long and the nucleotide variability is rather higher than the other NTSs. As has been mentioned before, the 5S rDNA type δ is probably a pseudogene, and neither the coding region nor the external promoters are bearing selective pressures that are homogenizing the array; therefore it is free to overcome mutations, thus increasing the nucleotide variability. The ribosomal genes (18S, 5.8S and 5S) showed lower levels of variability than the snRNA genes (except in the type δ of the 5S), thus indicating that the homogenizing forces of concerted evolution are more efficient in the ribosomal loci. Despite the nucleotide variability observed and the presence of various indels in the U2-U5 cluster, the different clones maintain a considerable homogeneity in their sequences, and no variants and pseudogenes were observed. Therefore, both major ribosomal and snRNA genes evolve according to the concerted model.

The karyotype obtained in *P. mediterraneus* shares the same karyotype formula (2n = 48; FN = 48) of most of the species of the Haemulidae family [54, and references therein]. Traditionally, it has been postulated that this karyotype formula is the ancestral (plesiomorphic) formula for the Teleostei subclass [73]; nevertheless, [74] suggested that the 2n = 48 acrocentric chromosome formula should have arisen later, during teleost spreading, and probably was the plesiomorphic karyotype for the Clupeomorpha and Euteleostei superorders. From the FISH analysis three chromosomal markers were obtained for the *P. mediterraneus* chromosome complement, due to the non-syntenic organization of the three probes used. Obtaining chromosomal markers is particularly important for distinguishing unequivocally chromosomal pairs from karyotypes composed of very small chromosomes and with similar sizes between adjacent pairs.

## Conclusions

Novel and surprising findings have been presented in the present survey, some of which have never been reported, such as the possible 5S rDNA horizontal transfer between two distantly-related species, the rubberlip grunt (*P. mediterraneus*) and the starry ray (*R. asterias*), and the genetic linkage between one 5S rDNA type and a putative tRNA-derived SINE. The occurrence of both the HT event and the SINE could have important evolutionary consequences; it is thus suggested that an exhaustive analysis of the Haemulidae family could provide interesting clues about the timing of the HT and SINE linkage and the consequences of these events. Further, such findings offer new and important clues concerning the organization and evolution of the 5S rDNA in fishes.

The variability observed in 5S rDNA does not correlate with the conservative organization of both major ribosomal gene and U2 snDNA, which indicates that the concerted model maintains the sequence homogeneity and drives the evolution of these two multigene families. The ITS-1 and U2 snDNA sequences have been studied for the first time in one species of the Haemulidae family. The result obtained with the ITS-1 sequence proves that the taxonomy of the Haemulidae family needs to be revised, and the ITS-1 could be a good molecular marker to this end. Moreover, the U2-U5 linkage could help to consolidate the Haemulidae taxonomy by analyzing the presence of this linkage in the different lineages of the family.

In addition, a preliminary physical map of the chromosome complement has been obtained, in which the three genes used (5S rDNA, 18S rRNA gene and U2 snDNA) are localized in three different chromosomal pairs. Hence, three chromosomal markers have been obtained, which is essential for a species with such small chromosomes.

## Methods

### Sampling and DNA extraction

Six specimens were collected to perform the molecular study, three from natural populations of the Southwestern coast of the Iberian Peninsula, and three obtained from the IFAPA “El Toruño” Center for aquaculture research (Cádiz, Spain). Larvae aged from 0 to 2 days (post-hatching) were used for cytogenetic analysis.

The *Fast DNA® Kit* (MP Biomedicals, Illkrich, France) was used to extract the DNA from the muscle tissue, following the manufacturer’s protocol. Extraction quality was validated by electrophoresis in agarose gel (1.5%) containing 0.5 μg ml^-1^ ethidium bromide.

### PCR amplification, cloning and sequencing

The 5S rDNA (including the NTS) and ITS-1 sequences were obtained by PCR using the primers described by [75] and by [76], respectively. The U2 snDNA was also obtained by PCR using the forward primer U2ang-Fwd (5’-CAAAGTGTAGTATCTGTTCTTATCAGC-3’) and the reverse U2ang-Rev (5’-CTTAGCCAAAAGGCCGAGA-3’). The reaction mix was according to [11]. The PCR mix was subjected to an initial denaturalization step at 94°C during 5 minutes, followed by 35 cycles of a denaturalization step at 94°C during 45 seconds, an annealing step at 57°C during 1 minute, and a elongation step at 72°C during 1.5 minutes. A final elongation step was carried out during 10 minutes at 72°C.

The PCR products were purified using the *NucleoSpin® Extract II kit* (Macherey-Nagel), and cloned into *pGEM®-T Easy Vector* (Promega, Madison, USA). Positive clones were grown in LB and plasmid DNA was extracted using *NucleoSpin® Plasmid kit* (Macherey-Nagel, Düren, Germany). DNA sequencing was performed with fluorescence-labeled terminator (*BigDye Terminator 3.1 Cycle Sequence kit*; Applied Biosystems, Carlsbad, USA) in an ABI3100 Genetic Analyzer.

### Sequence analysis

The sequences used in this study have been deposited in GenBank under the Accession Numbers from JN850618 to JN850660, and from JQ728001 to JQ728009. A total of 25 clones from 4 specimens were sequenced for the 5S rDNA (including NTS), 14 and 13 clones from 3 specimens were sequenced for the ITS-1 sequence and U2 snDNA gene respectively.

The sequence alignment was carried out using the ClustalX program [77]. The DnaSP version 5 program [78] was used to obtain the nucleotide variability (π) of both coding regions and spacers. Evolutionary distance between coding 5S rRNA types were calculated using the MEGA v5 program [79], by means of the number of base substitutions per site averaged over all sequence pairs, using the Kimura-2-parameter in relation to evolutionary distances (d). The sequences were also submitted to a BLASTN [80] search at the NCBI database to find sequence similarity with other species. Additionally, the CENSOR program [81] was used to search repetitive elements inside spacers.

To generate secondary structures, the sequences were submitted to two programs: RNAstructure 5.2 [82] and tRNAscan-SE [83]. In the first program, 15°C was selected to fold the fish sequences, and 37°C for the mammal species. Meanwhile, default parameters were left in place when the tRNAscan-SE program was run.

### Cytogenetic techniques

To obtain the chromosome preparations, the larvae were maintained during 3 hours in sea water with 0.015% colchicine. After this, the larvae were subjected to a hypotonic shock in 0.4% KCl, and finally were fixed in Carnoy solution (Ethanol: Acetic acid; 3: 1). A cellular suspension was obtained by homogenizing 3 larvae in 45% acetic acid and 3–4 drops were splashed onto clean slides heated at 45°C. To obtain the karyotype of *P. mediterraneus*, the slides were left to stain in 4% Giemsa solution and rinsed with distilled water.

Prior to carrying out the FISH techniques, the slides were pre-treated with RNAse, Pepsin, and fixed in formaldehyde, according to [84]. The probes were labeled by the PCR method with digoxigenin-11-dUTP (Roche Molecular Biochemicals, Barcelona, Spain) or tetramethylrhodamine-5-dUTP (Roche Molecular Biochemicals, Barcelona, Spain). The previously-mentioned primers were used, except for the major ribosomal cluster which was done by labeling a partial sequence of 18S rRNA gene with the primers described by [85]. Hybridization and post-hybridization methods were carried out according to [65]. The digoxigenin-labeled probes were detected by three incubations of 30 min each at 37°C in the order of the following immunological reagents: mouse anti-digoxigenin (Roche Molecular Biochemicals, Barcelona, Spain), rabbit anti-mouse-FITC (fluorescein isothiocyanate) (Sigma, Madrid, Spain) and goat anti-rabbit-FITC (Sigma, Madrid, Spain). Finally, better images were obtained with an epifluorescence microscope (Axioskop 2 Plus, Zeiss, Jena, Germany), equipped with a cooled camera (CoolSnap, Photometrics^©^ Inc., Tucson, USA).

## Authors’ contributions

MAM carried out the molecular genetic studies, the sequence alignment and analysis, the cytogenetic techniques, and drafted the manuscript. TP participated in the 5S rDNA molecular studies and the cytogenetic techniques. SPB participated in the 5S rDNA molecular study. IC participated in the sequence and cytogenetic analysis of the study, discussed the results and helped to draft the manuscript. MM carried out the sampling, tissue extraction and larval maintenance. LR conceived and coordinated the study, participated in its design, discussed the results and corrected the manuscript. All authors read and approved the final manuscript.
